# Effects of chocolate containing *Leuconostoc mesenteroides* strain NTM048 on immune function: a randomized, double-blind, placebo-controlled trial

**DOI:** 10.1186/s12979-018-0139-2

**Published:** 2018-11-20

**Authors:** Reiko Kuroda, Hiroaki Higuchi, Keishirou Yoshida, Yasunori Yonejima, Keiko Hisa, Masanori Utsuyama, Kenji Osawa, Katsuiku Hirokawa

**Affiliations:** 1 Central Laboratory, LOTTE Co., Ltd., 1-1, Numakage 3-chome, Minami-ku, Saitama, 336-8601 Japan; 2 Nitto Pharmaceutical Industries, Ltd., 35-3, Minamibiraki, Kamiueno-cho, Muko, Kyoto, 617-0006 Japan; 3 Institute for Health and Life Science, Tokyo Medical & Dental University, 3-10, Kandasurugadai 10-chome, Chiyoda-ku, Tokyo, 101-0062 Japan; 4 Department of Comprehensive Pathology, Tokyo Medical & Dental University, 1-5-45, Yushima, Bunkyo-ku, Tokyo, 113-8510 Japan

**Keywords:** *Leuconostoc mesenteroides* strain NTM048, Immunological parameters, Scoring of immunological vigor, Chocolate, Randomized controlled trial

## Abstract

**Background:**

Previous reports showed that oral administration of *Leuconostoc mesenteroides* strain NTM048 increases IgA levels and CD4+ T cell population in feces and mice, respectively, as revealed by flow cytometric analysis of splenocytes. This study aimed to evaluate the effect of chocolate supplemented with *L. mesenteroides* strain NTM048 (> 1.00 × 10^9^ CFU/day, NTM048) on the immune parameters of healthy subjects, using a randomized, placebo-controlled, double-blinded study design.

**Methods:**

Participants (mean age: 46.3 years) ingested 28 g of test food daily, at a time of their own choice, for 4 weeks. The immunological parameters of all participants were evaluated two times (pre- and post- ingestion). At the end of the study, various immunological parameters of the participants were measured and scoring of immunological vigor (SIV) was performed using a comprehensive algorithm.

**Results:**

Ingestion of NTM048-supplemented chocolate significantly improved SIV in the NTM048 group (18.6 ± 1.6) compared to that in the placebo group (17.8 ± 2.0) after 4 weeks (*p* = 0.049). Several immunological parameters (CD8^+^T cells, CD8^+^CD28^+^ T cells, and memory T cells) were significantly elevated in the NTM048 group as compared to the placebo group (all *p* < 0.05). In addition, T cell proliferation index at post-ingestion significantly increased compared with that at pre-ingestion in the NTM048 (*p* = 0.017) and placebo groups (*p* = 0.037), although no differences were observed between the two groups.

**Conclusion:**

Our results suggest that ingestion of chocolate supplemented with NTM048 is effective against the age-related decline in T cell-related immune functions.

**Trial registration:**

UMIN Clinical Trials Registry UMIN000021989. Registered 19 April 2016, https://upload.umin.ac.jp/cgi-open-bin/ctr_e/ctr_view.cgi?recptno=R000025321

## Background

Depressed immune activity in elderly people is one of the major factors responsible for the development of the top three diseases - infections, cancers, and vascular diseases. Depression of immune activity mainly occurs in T cells starting as early as 20 years of age, showing a decline of 50% of the peak level in some individuals at the age of 40 years. Thus, slowing the rate of the age-related decline of immune activity is expected to be significant for prolonging a healthy life-span.

In this study, we tested a novel strategy to recover the depressed immune activity in humans. Lactic acid bacteria are one of the most important microorganisms present in fermented foods such as yogurt, cheese, miso, and pickles. Ilya Ilyich Mechnikov, who was awarded the 1908 Nobel Prize in Physiology or Medicine in recognition of his work on immunity, advocated a theory that aging is caused by toxic bacteria in the intestine and that lactic acid can increase life expectancy. Since then, a lot of researchers have been interested in the functionality of lactic acid, and many studies have demonstrated the physiological effects of lactic acid [1, 2].

Recent reports have indicated that metabolic products from lactic acid bacteria, such as exopolysaccharides (EPS) and fatty acids, have significant beneficial effects on human health. For example, dietary linoleic acid is converted into conjugated linoleic acid by lactic acid bacteria [3]. Conjugated linoleic acid is known to reduce systemic inflammatory mediators in healthy young adults [4], and to improve glycemic response, lipid profile, and oxidative stress in obese patients [5]. Conjugated linoleic acid can be synthesized by chemically isomerizing linoleic acid derived from safflower oil, but various by-products are also produced at the same time. Recent studies reported that linoleic acid is converted to conjugated linoleic acid by *Lactobacillus plantarum* and *Pediococcus acidilactici*. *Lactobacillus casei* strain Shirota and *Lactococcus lactis* YIT2027 have high glutamic acid decarboxylase and high protease activities. Using mixed culture of these two microbes, we obtained fermented milk containing γ-aminobutyric acid, which has a significant hypotensive effect in mildly hypertensive individuals [6]. *Lactobacillus helveticus* produces valine-proline-proline and isoleucine-proline-proline, which are hypotensive peptides [7, 8].

EPS have been reported to enhance immune functions [9]. Kefiran, which consists of glucose and galactose, is one of the EPS produced by *Lactobacillus kefiranofaciens*. Oral administration of kefiran has been reported to increase macrophage counts and levels of immunoglobulin A in the lamina propria of the intestinal mucosa [10]. The acidic EPS produced by *Lactobacillus delbrueckii* ssp. *bulgaricus* OLL 1073R-1 may also affect immune parameters. In a previous report, oral administration of acidic EPS was shown to enhance natural killer cell activity in mouse spleen cells [11]. Ingestion of yogurt fermented with *L. bulgaricus* OLL1073R-1 was reported to increase NK cell activity in healthy participants and induce resistance to common cold [12].

*Leuconostoc mesenteroides* strain NTM048 (NTM048) was originally isolated from green peas. In mice, oral administration of food containing NTM048 for 2 weeks increased IgA levels in feces [13]. The EPS produced by NTM048 are speculated to be comprised mainly of glucose and fructose. A previous study investigated the effects of NTM048 EPS intake on the systemic immune system in mice and found that CD3^+^ T cell and CD4^+^ T cell populations increased in the spleen [14]. The results suggested that ingestion of NTM048 EPS exerts immunostimulatory effects in mice. However, the effects of NTM048 ingestion on the human immune system remain unknown. In a previous study, we reported that *Lactobacillus brevis* NTT001 can survive transit through the gastrointestinal tract when ingested via chocolate; thus chocolate can be useful tool to protect probiotics [15]. Through fecal analysis, we revealed that chocolate containing *L. brevis* NTT001 improves the intestinal environment by increasing the level of acetic acid and number of *Lactobacillus* in the intestine [16].

On the other hand, there are many reports on the biological effects of cacao. For example, cacao polyphenol and ω-3 fatty acids are effective in decreasing the risk of cardiovascular diseases [17]. Flavonoids are much richer in cacao than other plants and have an antioxidant potential, decreasing the risk of diabetes by influencing insulin resistance. Furthermore, phytochemicals in cacao are effective in the amelioration of skin-aging [18]. In this study, we aimed to clarify the effects of NTM048-supplemented chocolate on the immune parameters of healthy subjects. In addition, we tested the safety of NTM048-supplemented chocolate by physical examination, urinalysis, and blood tests.

## Results

### Participant characteristics and flow

**Fig. 1:**
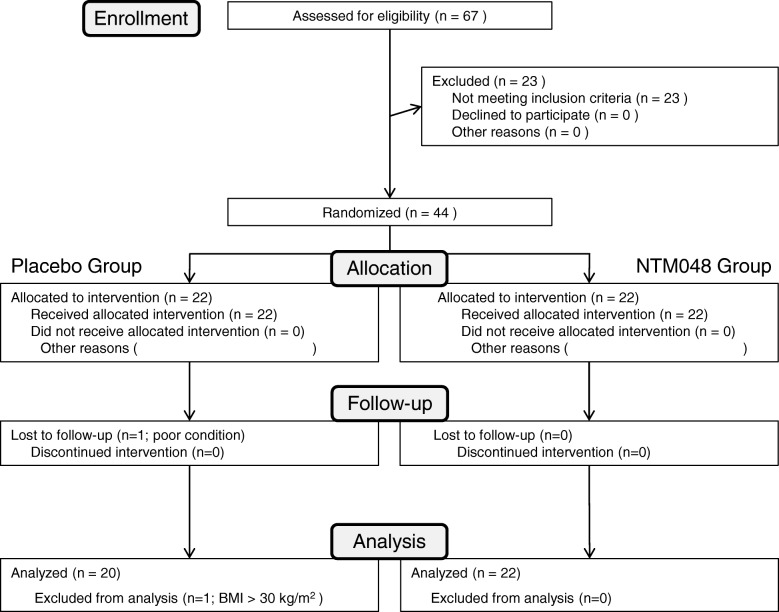
Flow and number of subjects in each phase of the study. Forty-four of the 67 participants were randomized at pre-ingestion; 22 in the NTM048 group and 20 in the placebo group were included in the final analysis

Figure 1 shows the participant flow diagram. Sixty-seven volunteers were initially recruited. They were pronounced healthy by routine health checkup, and serum biochemical data were within normal range. Among them, 42 subjects who showed relatively lower immunity score were selected for this study. They were grouped into two groups: - placebo group and NTM048 group. The participants ingested 28 g of the test food (either the experimental food or the placebo) per day at a time of their own choosing for 4 weeks, after which the peripheral blood was obtained for immunological analysis. Table 1 shows the characteristics of the two groups.

**Table 1:** Group Participant characteristics

	Placebo (*n* = 20)	NTM048 (*n* = 22)
Age(years)	45.8 ± 9.9	46.8 ± 11.6
Range(years)	30–69	21–63
Male/female(n)	3/17	5/17
Height (cm)	159.7 ± 7.4	161.8 ± 6.3
Body weight (kg)	52.1 ± 10.1	53.9 ± 9.4
Body mass index (kg/m^2^)	20.4 ± 3.2	20.5 ± 2.6
Body fat percentage (%)	21.8 ± 6.8	21.4 ± 5.5
Systolic blood pressure (mmHg)	111.2 ± 10.0	115.8 ± 17.2
Diastolic blood pressure (mmHg)	70.9 ± 5.8	74.2 ± 12.1
Heart rate (bpm)	69.1 ± 8.7	70.5 ± 8.6

Values are shown as means ± SD

Group participant characteristics (age, body weight and so on) are shown as means and standard deviations

### Effect on scoring of immunological vigor (SIV)

SIV (immunity score) at pre- and post-ingestion are shown in Table 2A. SIV does not represent the level of a single immunological parameter, but is assessed using 8 immunological parameters, suggesting that SIV strongly reflects the comprehensive level of the immunological activity of a subject.

**Table 2:** (a-e) Changes in immunological parameters after NTM048 ingestion

Item	group	pre-ingetion	post-ingestion	*p* value	
A					
immunity score	Placebo	17.8 ± 1.9	17.8 ± 2.0	1.000	0.049^#^
	NTM048	17.7 ± 1.9	18.6 ± 1.6	0.015^*^	
B					
CD8^+^ T cells (/μL)	Placebo	261 ± 98	264 ± 126	0.859	0.047^#^
	NTM048	234 ± 125	293 ± 196	0.034^*^	
CD8^+^CD28^+^ T cells (/μL)	Placebo	197 ± 72	197 ± 99	0.981	0.045^#^
	NTM048	152 ± 67	179 ± 71	0.007^*^	
memory T cells (/μL)	Placebo	468 ± 122	469 ± 123	0.938	0.022^#^
	NTM048	477 ± 146	543 ± 166	0.006^*^	
C					
lymphocytes (/μL)	Placebo	1517 ± 283	1574 ± 327	0.238	0.094
	NTM048	1549 ± 371	1835 ± 604	0.020^*^	
CD3^+^ T cells (/μL)	Placebo	1113 ± 261	1154 ± 280	0.275	0.083
	NTM048	1104 ± 239	1317 ± 507	0.022^*^	
CD4^+^ T cells (/μL)	Placebo	748 ± 219	762 ± 225	0.560	0.094
	NTM048	768 ± 243	841 ± 231	0.020^*^	
T-lymphocyte age (years)	Placebo	52.6 ± 9.2	52.8 ± 9.9	0.815	0.085
	NTM048	55.3 ± 8.8	54.0 ± 9.3	0.025^*^	
Immunological age (years)	Placebo	52.5 ± 10.0	50.7 ± 9.4	0.131	0.511
	NTM048	52.6 ± 11.5	49.9 ± 12.7	0.009^*^	
D					
T cell proliferative activity	Placebo	1.38 ± 0.2	1.51 ± 0.2	0.047^*^	0.804
	NTM048	1.46 ± 0.2	1.53 ± 0.2	0.085	
T-cells proliferation index (TCPI)	Placebo	1.56 ± 0.5	1.77 ± 0.6	0.037^*^	0.352
	NTM048	1.62 ± 0.5	2.01 ± 0.8	0.017^*^	
E					
Neutrophils (/μL)	Placebo	2976 ± 860	2775 ± 731	0.380	0.941
	NTM048	2554 ± 923	2757 ± 816	0.113	
Naïve T cells (/μL)	Placebo	280 ± 119	293 ± 130	0.265	0.859
	NTM048	291 ± 131	298 ± 121	0.714	
B cells (/μL)	Placebo	230 ± 64	218 ± 65	0.269	0.040^#^
	NTM048	265 ± 134	289 ± 136	0.163	
NK cells (/μL)	Placebo	166 ± 71	187 ± 70	0.066	0.363
	NTM048	200 ± 121	262 ± 258	0.257	
CD4/CD8 T cells ratio	Placebo	3.47 ± 2.2	4.54 ± 5.2	0.156	0.118
	NTM048	5.79 ± 7.6	4.44 ± 4.4	0.139	
Naive/Memory T cell ratio	Placebo	0.60 ± 0.2	0.63 ± 0.2	0.377	0.166
	NTM048	0.63 ± 0.3	0.58 ± 0.2	0.228	
immunity grade	Placebo	2.80 ± 0.4	2.80 ± 0.5	1.000	0.302
	NTM048	2.82 ± 0.4	2.95 ± 0.5	0.186	
NK cells activity (%)	Placebo	48.0 ± 19.0	46.6 ± 16.6	0.452	0.470
	NTM048	48.9 ± 13.4	50.1 ± 16.4	0.754	
IFN-γ (pg/mL)	Placebo	13.5 ± 41.8	22.3 ± 83.4	0.359	0.345
	NTM048	3.12 ± 3.4	3.64 ± 4.1	0.164	

Values are mean ± SD

*:*p* < 0.05, comparison between pre-ingestion and post-ingestion, paired *t*-test

#:*p* < 0.05, comparison between groups using Student’s t-test or analysis of covariance (ANCOVA) depending on distribution of data

In the NTM048 group, immunity score significantly increased from pre-ingestion value of 17.7 ± 1.9 to post-ingestion value of 18.6 ± 1.6 (*p* = 0.015). Contrastingly, SIV did not differ between pre- (17.8 ± 1.9) and post-ingestion (17.8 ± 2.0) in the placebo group (*p* = 1.000). When compared between groups, the NTM048 group showed a significantly higher SIV than the placebo group post-ingestion (*p* = 0.049).

### Effect on immunological parameters

The number of CD8^+^T cells, CD8^+^CD28^+^ T cells, and memory T cells increased significantly at post-ingestion of NTM048 (*p* = 0.0334, *p* = 0.007 and *p* = 0.006, respectively) (Table 2B), but not in the placebo group. In addition, these levels at post-ingestion showed a statistically significant difference between NTM048 and placebo groups (*p* = 0.047, *p* = 0.045, and *p* = 0.022 respectively).

The populations of lymphocytes, CD3^+^ T cells, and CD4^+^ T cells increased significantly between pre-ingestion and post-ingestion in the NTM048 group (*p* = 0.020, *p* = 0.022, *p* = 0.020, respectively) (Table 2C). T-lymphocyte age decreased significantly between pre-ingestion (55.3 ± 8.8) and post-ingestion (54.0 ± 9.3) in the NTM048 group (*p* = 0.025). In contrast, in the placebo group, T-lymphocyte age at pre- and post-ingestion was 52.6 ± 9.2 and 52.8 ± 9.9, respectively, and no significant difference was observed. Similarly, the immunological age of the NTM048 group at pre-ingestion was 52.6 ± 11.5 years and was significantly decreased to 49.9 ± 12.7 years at post-ingestion (*p* = 0.009). However, no significant difference in immunological age was observed in the placebo group and between the two study groups.

Table 2D shows T cell proliferative activity and T cell proliferation index (TCPI). T cell proliferative activity at post ingestion showed a significant increase in the placebo group (*p* = 0.047), but only a slight increase was observed in the NTM048 group (*p* = 0.085). A significant increase in T cell proliferation index was observed in both NTM048 and placebo groups (*p* = 0.037 and *p* = 0.017, respectively). However, when compared between groups, no statistical significance was observed.

The population of neutrophils, naïve T cells, B cells, NK cells, the percentage of CD4/CD8 ratio, naive T/ memory T cell ratio, immunity grade, NK cell activity, and IFN-γ showed no significant differences between pre-ingestion and post-ingestion in any of the groups (Table 2E).

### Safety assessment

Safety of NTM048-supplemented chocolate was assessed by routine health check-up including examination of urine and peripheral blood before and after the injection. The results showed that the level of inorganic phosphorus increased in both placebo and experimental groups; however, this slight elevation could be ignored considering that all the other data obtained by health-check-up were within normal range.

## Discussion

This study aimed to evaluate the effect of NTM048-supplemented chocolate on the immune parameters of healthy subjects. The experimental group showed a significant increase in the populations of lymphocytes, CD3^+^ T cells, and CD4^+^ T cells after ingestion of the chocolate (Table 2C). Only a slight difference was observed between the NTM048 group and the placebo group with *p* values of 0.094 in lymphocytes and CD4^+^ T cells, and 0.083 in CD3^+^ T cells. Although, the p values are slightly out of the significance range, it may be considered as a trend towards significance. However, three important T cell subpopulations (CD8^+^ T cells, CD8^+^CD28^+^ T cells, and memory T cells) showed significant increases during post-ingestion measurements and showed a significant difference between NTM048 and the placebo groups. In addition, SIV (immunity score), which was calculated using a comprehensive algorithm [19–21], showed a significant increase at post-ingestion measurement between NTM048 and the placebo groups. The immunity score is calculated based on eight immunological parameters and it reflects the immunological function at the whole-body level. These results are promising, and NTM048-supplemented chocolate could be beneficial for immunological function in humans.

T lymphocyte age and immunity age are easy-to-understand indices that show the level of immunity. T lymphocyte age is calculated using the CD8^+^CD28^+^ T cell number, and immunity age is calculated from the T cell proliferation index. Both T lymphocyte age and immunity age showed a significant decrease after ingestion of NTM048-supplemented chocolate. A lower T lymphocyte age represents a younger immune system. T cell proliferative activity is a more relevant measure of the immune system from a functional perspective. T cell proliferative activity is based on in vitro data and is calculated using an adjusted number of lymphocytes and does not directly reflect the whole-body immune activity level. To extrapolate the T cell proliferative activity to the whole-body level, we devised a new parameter - the TCPI [19], which is calculated using in vitro T cell proliferative activity and the number of T cells in peripheral blood. In the present study, TCPI was significantly increased in both NTM048 and placebo groups, but the level was higher in NTM048 than in the placebo group, although it was not statistically significant. This indicated that chocolate by itself can improve the TCPI. This may be attributed to its anti-oxidant properties.

NTM048 is a lactic acid bacterium isolated from green peas. This species produces large amounts of exopolysaccharides (EPS). Increased proliferation of CD4^+^ T cells has been reported in mice fed NTM048-derived EPS [14]. A supplementary analysis revealed that both CD4^+^ T and CD8^+^ T cell counts increased significantly after 4 weeks of NTM048 consumption. Variation in CD4^+^ T cell counts was similar to those reported in earlier studies. This study elucidated the increase of not only CD4^+^T cells but also of CD8^+^ T cells. Immunologically, CD8^+^CD28^+^ T cells, when activated, destroy virus-infected cells and tumor cells. Thus, an increase in this population is of great significance in strengthening immunity. CD4^+^ T cells belong to the category of helper T cells (Th cells), with their major types being Th1, Th2, Th17, and regulatory T cells (Treg). They are responsible for cellular immunity in general, allergic inflammation, and infection control [22, 23]. We also measured the amount of interferon -γ produced by Th1 cells. However, in the present study, IFN-γ measurements were below the detection level and no significant results were obtained. More detailed experiments including in vitro culture of lymphocytes and measurements of cytokine production would be required to study the changes in helper T cells.

Finally, we tested the safety of NTM048-supplemented chocolate by performing physical examinations, urinalysis, and blood tests before and after 4 weeks of ingestion of the experimental food. Although some parameters fluctuated significantly within groups, all scores except for inorganic phosphorus fluctuated were within the reference value, and no medical problems were noted. The mean values of inorganic phosphorus were significantly above the reference value in both groups after 4 weeks of feeding. It is possible that the subjects were dehydrated at the time of blood collection, but this could not be verified because the amount of water ingested in the 6-h period prior to the examination was not recorded. All subjects were judged to be healthy by the doctor. We conclude that the consumption of NTM048-supplemented chocolate for 4 weeks may improve immune system function. In addition, NTM048-supplemented chocolate was found to be safe for consumption under the test conditions.

The strengths and limitations in this study are as follows: this study showed that ingestion of chocolate containing NTM048 may enhance immune activity. Foods containing viable lactic acid bacteria need to be stocked at a low temperature, whereas chocolate containing NTM048 can be ingested anywhere and anytime. On the other hand, this study mainly recruited middle-aged participants, and thus, it would be useful to investigate the effect of NTM048-containing chocolate in elderly people with depressed immune activity.

The limitation of this study is that there were few male participants. Nevertheless, by increasing the sample size and conducting research on elderly people in the future, this food may contribute to prolonging a healthy life-span.

## Conclusions

This study aimed to evaluate the effect of chocolate supplemented with NTM048 (> 1.00 × 10^9^ CFU/day) on the immune parameters of healthy subjects. A significant immunological improvement was observed in the NTM048 group compared to the placebo group after 4 weeks of consumption of the experimental food. Our results suggest that ingestion of NTM048-supplemented chocolate may improve immunological functions in humans by activating the T cells. NTM048-supplemented chocolate was found to be safe for consumption in our safety studies. Therefore, NTM048-supplemented chocolate could be used for immune restoration.

## Methods

### Study design

A randomized, double-blind, parallel group, placebo-controlled trial was conducted with two groups of healthy subjects between June 27 and August 9, 2016 (4 weeks) in Tokyo, Japan. Participants were assigned randomly to the placebo group (chocolate not containing *L. mesenteroides)* or to the experimental group (chocolate supplemented with *L. mesenteroides* strain NTM048, NTM048 group) at a 1:1 ratio. The participant allocation table was blinded until all analyses were completed and the data set was locked.

### Test food

The experimental food was prepared by infusing NTM048 (> 1.00 × 10^9^ CFU/day, obtained from Nitto Pharmaceutical Co., Ltd.) into chocolate. The placebo chocolate was prepared exactly as the NTM048-supplemented chocolate was prepared, except that NTM048 was not added, such that it was indistinguishable from the experimental food in appearance, taste, and flavor. Participants ingested 28 g of test food (either the experimental food or the placebo food) per day at a time of their own choosing.

### Participants

This study was conducted in compliance with the Declaration of Helsinki (2013) and the Ethical guidelines for medical and health research involving human subjects (2014). Written informed consent was obtained from the participants. The study protocol was reviewed and approved by the institutional ethical review boards at Seishin-kai Medical Association Inc., Takara Clinic. The outline of this study was registered in the UMIN clinical trials registry as UMIN000021989 (https://upload.umin.ac.jp/cgi-open-bin/ctr_e/ctr_view.cgi?recptno=R000025321).

Sixty-seven healthy volunteers, who were 20 years or older, were recruited to participate in this study by Orthomedico Inc. (Bunkyo-ku, Tokyo). In total, 44 participants (35 females and 9 males, 47.0 ± 11.2 years old.) with relatively low immunity scores (scoring immunological vigor, SIV: between 13/24 and 23/24) were included.

All participants were judged to be eligible for participation in the study by a doctor. The participants were divided into two groups using the Statlight System #11, Yukms, Tokyo, Japan by Orthomedico Inc. (Bunkyo-ku, Tokyo). The exclusion criteria were as follows: a) persons who have a medical history of cancer, heart failure, or cardiac infarction, b) persons who use dietary supplements or pharmaceuticals (including Chinese medicine) regularly, c) persons who consume food that affect the intestinal microbiota, such as prebiotic/probiotics (e.g., *Bifidobacterium*, *Lactobacillus*, oligosaccharides, fiber) and yogurt at least three times per week, d) persons who consume beverages and foods rich in lactic acid bacteria, foods containing viable bacteria such as bifidus bacteria and *Bacillus natto*, fiber-rich foods, or those rich in oligosaccharides, e) persons who consume foods, such as “Foods for Specified Health Uses” or “Foods with Functional Claims”, that affect the intestinal microbiota, at least three times a week, f) persons with food allergies, g) persons who are pregnant, breast-feeding, or plan to become pregnant, h) persons who had participated in another clinical trial in the 3 months prior to signing the informed consent form for this trial, i) others considered inappropriate for the study by the physician. The compliance criteria during the test were as follows: a) to ingest the stated dosage of the test food per the administration criteria, b) not to change eating habits or lifestyle and avoid excessive drinking or eating, c) not to ingest daily supplements, such as oligosaccharides and so on, d) not to engage in consumption of alcohol or excessive exercise before the examination, e) not to eat or drink anything except water for 6 h before blood sample collection, f) in case of a change in physical condition during the test, to contact the research institution immediately and to follow their instructions regarding the test.

### Procedure

Participants ingested 28 g of the test food per day at a time of their own choice every day for 4 weeks. All participants visited the Takara Clinic two times (pre- and post- ingestion) and were given a routine health check. All participants answered a daily questionnaire during the intake period and submitted their responses once a week.

### Primary outcome

The primary outcome was measured using the scoring of immunological vigor (SIV) algorithm developed by Hirokawa et al. [17–19]. The SIV is a comprehensive scoring system for evaluating human immune function using the following eight immunological parameters: T cell counts, ratio of CD4^+^/CD8^+^ T cells, naïve-T cell counts, ratio of naïve/memory T cells, B cell counts, NK cell counts, CD28^+^ T cell counts within CD8^+^ T cells (CD8^+^CD28^+^ T cell), and TCPI. Each parameter is scored using 3 grades (score 1 “low immunity level”; score 2 “moderate immunity level”; and score 3 “high immunity level”) based on a database created by Hirokawa et al. (19). The total score for the 8 parameters is referred to as the SIV, ranging from 8 to 24. The SIV contains five grades; grade I is the critical zone (score, 8–11); grade II is the warning zone (score, 12–15); grade III is the observation zone (score, 16–19); grade IV is the safety zone (score, 20–23); and grade V is a sufficiently high level of immunity (score, 24).

### Secondary outcomes

The secondary outcomes were as follows: T cell counts, ratio of CD4^+^/CD8^+^ T cells, naïve T cell counts, ratio of naïve/memory T cells, B cell counts, NK cell counts, CD8^+^CD28^+^ T cell counts, T cell proliferative activity, TCPI, T lymphocyte age, immunological age, NK cell activity, and INF-γ. The number of CD8^+^CD28^+^ T lymphocytes is inversely correlated with actual age [24]. T lymphocyte age is determined by substituting CD8^+^CD28^+^ T lymphocytes counts into the following relevant relational expression:

T lymphocyte age = (523 - CD8^+^CD28^+^ T lymphocyte count)/4.87.

In addition, since TCPI is inversely correlated with actual age, the immunological age can be determined by substituting the coefficient into the following relational expression [19]:

Immunological age = (2.535 - TCPI) / 0.0174.

The ages calculated using the above theory are compared with the actual age, and the functional level of the immune system is estimated. TCPI is a coefficient calculated from T cell counts and T cell proliferative activity. TCPI indicates the proliferation ability of T cells at an individual level. A higher TCPI value suggests a higher resistance to infection.

### Safety assessment

Safety assessments were conducted to determine whether ingestion of the test food posed a risk of developing any clinical condition in the participants. Safety assessments involved measurement of the following parameters: 1) Physical examination (height, weight, BMI, body fat percentage, blood pressure, systolic blood pressure, diastolic blood pressure, heartbeat), 2) Urinalysis (protein, glucose, urobilinogen, bilirubin, ketone bodies, pH, occult blood), 3) Peripheral blood test (AST (GOT), ALT (GPT), γ-GTP, ALP, LD(LDH), LAP, total bilirubin, direct bilirubin, indirect bilirubin, cholinesterase, zinc sulfate turbidity test (ZTT), total protein, urea nitrogen, creatinine, uric acid, creatine kinase, sodium, potassium, chlorine, calcium, inorganic phosphorus, serum iron, total cholesterol, HDL-cholesterol, LDL-cholesterol, triglycerides, free fatty acids, glucose, hemoglobin A1c, glycated albumin).

### Participant diary

The participants kept a diary from the beginning of the study until the day before the final examination, noting the following information: the number of times they consumed the test food, menstruation period, physical condition (good, normal, poor), and whether they consumed food containing lactic acid bacteria (the presence or absence, amount, product name), change in physical/living conditions, and whether or not they were taking any pharmaceuticals.

### Sample size estimation and statistical analysis

To evaluate SIV, a minimum sample size of 20 subjects per group was determined through a power analysis with the following assumptions: a statistical power of 80%, a significance level of 5%, an effect size of 0.85, and an allocation ratio of 1:1. Comparison of outcomes between groups was performed using Student’s t-test or analysis of covariance (ANCOVA) according to the distribution of data. After confirming the parallelism and significance of the regression, we performed an ANCOVA, including the baseline value as a covariate value. Data analyses were performed using IBM SPSS Statistics (version 23.0 for Windows, IBM Japan, Inc., Tokyo, Japan). The level of statistical significance was set at *p* < 0.05.

## Abbreviations

ANCOVA: Analysis of covariance
EPS: Exopolysaccharides
*L. mesenteroides*: *Leuconostoc mesenteroides*
SIV: Scoring of immunological vigor
TCPI: T-cell proliferation index
